# Ab Initio Quantum-Mechanical Predictions of Semiconducting Photocathode Materials

**DOI:** 10.3390/mi12091002

**Published:** 2021-08-24

**Authors:** Caterina Cocchi, Holger-Dietrich Saßnick

**Affiliations:** Physics Department, Carl von Ossietzky Universität Oldenburg, D-26129 Oldenburg, Germany; holger-dietrich.sassnick@uni-oldenburg.de

**Keywords:** photocathodes, semiconductors, density-functional theory, many-body perturbation theory, high-throughput screening

## Abstract

Ab initio Quantum-Mechanical methods are well-established tools for material characterization and discovery in many technological areas. Recently, state-of-the-art approaches based on density-functional theory and many-body perturbation theory were successfully applied to semiconducting alkali antimonides and tellurides, which are currently employed as photocathodes in particle accelerator facilities. The results of these studies have unveiled the potential of ab initio methods to complement experimental and technical efforts for the development of new, more efficient materials for vacuum electron sources. Concomitantly, these findings have revealed the need for theory to go beyond the status quo in order to face the challenges of modeling such complex systems and their properties in *operando* conditions. In this review, we summarize recent progress in the application of ab initio many-body methods to investigate photocathode materials, analyzing the merits and the limitations of the standard approaches with respect to the confronted scientific questions. In particular, we emphasize the necessary trade-off between computational accuracy and feasibility that is intrinsic to these studies, and propose possible routes to optimize it. We finally discuss novel schemes for computationally-aided material discovery that are suitable for the development of ultra-bright electron sources toward the incoming era of artificial intelligence.

## 1. Introduction

The generation of ultra-bright electron sources is currently demanded in many fields of applications, ranging from high-resolution spectroscopy [1] to electron diffraction [2] and electron microscopy [3]. Laser-driven photocathode radio-frequency injectors are currently employed in particle accelerators to pursue enhanced beam brightness, which in turn grants unprecedented opportunities for material characterization following the dynamics of charge carriers in their natural space and time scales [4]. In state-of-the-art photoinjectors, electron beam performance is largely dominated by the intrinsic emittance at the photocathode. Further developments in this direction demand substantial improvements of the electron sources. For decades, photocathodes were built of metals, such as copper [5], which are characterized by a large carrier density and low work function. However, these materials are unsuitable for the next generation of electron sources: their characteristic absorption in the ultra-violet region requires power-expensive frequency conversion, and their tendency to heat up represents an additional source of inefficiency [6]. New classes of semiconductors [7], such as Cs${}_{2}$Te [8,9] and multi-alkali antimonides [10,11,12,13,14,15,16,17,18,19,20,21], have therefore emerged in the last decade as photocathode materials.

**Figure 1:**
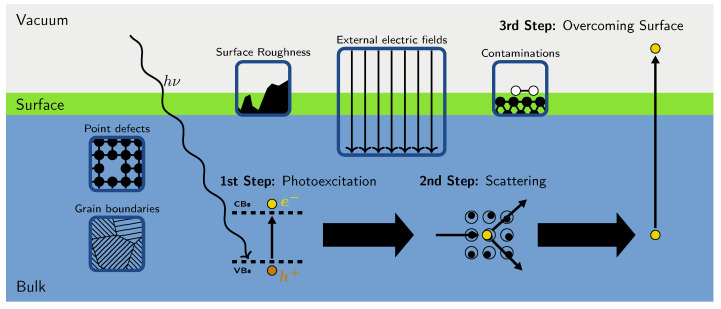
Schematic overview of the main processes involved in Quantum-Mechanical modeling of semiconducting photocathode materials from first principles. The main physical mechanisms in the bulk (**bottom**, blue) on the surface (**middle**, green), and in vacuo (**top**, light grey) are sketched in the boxes. On top of these illustrations, the three steps of photoemission are also outlined.

In spite of the great potential of the aforementioned families of compounds, and the efforts that have been made to control their synthesis and unravel their properties [18,22,23,24], this research area is still in its infancy. A number of challenges have to be tackled in order to gain sufficient insight to control and manipulate the composition of alkali antimonides and tellurides as well as their characteristics for photocathode applications (see Figure 1). To start, these materials pose severe issues in terms of stability, lifetime, and reproducibility of their growth procedures [18,25]. Their high surface reactivity makes them subject to atmospheric contamination, which should be reduced to a minimum to prevent deterioration as well as to enhance sample durability and photoemission yield [6,26,27]. Likewise, surface roughness can be detrimental for the lifetime and the performance of the materials [9,28,29,30,31]. Established growth methods for alkali-based photocathodes are based on sequential deposition or co-deposition techniques [12,32,33]. Although still largely based on empirical trial-and-error procedures, effective recipes have been developed to meet application requirements [9,18,34,35]. Yet, the resulting samples are often polycrystalline [12,32] whereby grain boundaries and point defects are additional sources for lifetime and performance degradation. Finally, in operational conditions, photocathodes are subject to (intense) electric fields, which can alter their properties. As all these external factors act simultaneously on the samples, it is extremely hard to disentangle their effects experimentally in order to substantially improve the materials and their performance.

Another major challenge in the search for more efficient materials for electron sources is related to the prediction of their photoemission performance. In semiconductors, which are characterized by a gap between occupied and unoccupied electronic bands, the ability of materials to eject photoelectrons [36] can be summarized in three steps [37] (see Figure 1). Initially, the system is impinged by a photon, which promotes an electron from an occupied band to an initially empty one. In this photoabsorption process, an electron-hole pair is formed and held together by the Coulomb attraction for a finite excitation lifetime. The generated charge carriers may undergo scattering events on their way to reach the surface. In semiconductors, this second step is typically dominated by electron–phonon coupling, since electron-electron scattering is much less pronounced than in metals, due to the lower charge carrier density. The third and final step entails the emission of the photoelectrons. Only particles with residual energy exceeding the work function of the material are able to leave the surface and be ejected into the vacuum. The three-step model has been employed for decades to fit empirical data and remains to date the method of choice to evaluate the emission yield of photocathodes [38,39,40]. This approach has, however, several limitations, and not only when it comes to predicting the performance of materials that have not been produced nor experimentally characterized yet. The presence of defects, contaminants, grain boundaries, etc., requires a huge amount of parameters that are hard to control [41].

A number of schemes have been proposed in the last few years toward a semi-empirical extension of the three-step model [42]. For example, Monte Carlo methods have been successfully employed to simulate scattering and transport processes [43,44,45]. More recently, the first attempt to feed the three-step model exclusively with ab initio results was proposed [46], showing the potential of this route. Yet, as the quality of first-principles results depends critically on the chosen approximations, it is essential to assess the trade-off between accuracy and computational efficiency [47,48]. Providing clear indications in this regard has become particularly important in light of the increasing number of non-expert users that approach ab initio methods.

Current applications of ab initio material modeling encompass a huge range of scientific fields, from astrochemistry [49] to photobiology [50], and from metallurgy [51] to geology [52]. The reason for this immense success is certainly related to the versatility of first-principles methods, which require only structural information and chemical composition as input in calculations, as well as to the excellent parallelizability and scalability of corresponding algorithms [53,54], which currently enable the treatment of realistic systems described by up to several thousands of electrons. Recently, ab initio methods were adopted also in the description of the electronic properties of photocathode materials, including both metals [55,56,57] and semiconductors [58,59,60,61,62,63,64,65,66,67,68,69,70]. These studies have shown the potential of first-principles approaches not only to predict their properties, but also to rationalize their physical behaviors. Given the challenges discussed above and visualized in Figure 1, ab initio calculations can relevantly contribute to gaining knowledge on alkali antimonides and tellurides, and complement experiments to establish efficient growth recipes and to assess the properties of the produced photocathodes.

In the following, we will discuss the role of Quantum-Mechanical ab initio methods for electronic-structure theory for the development of more efficient materials for the next generation of electron sources. To do so, we will first review the first-principles approaches that are most suitable to model the properties of semiconducting photocathode materials (Section 2). With the examples of Cs${}_{2}$Te and cesium antimonides, which are currently used and investigated in many experimental facilities worldwide, we will show the ability of these methods to determine material properties, thereby providing the key ingredients to evaluate photoemission performance based on the three-step model [37] (Section 3). Next, we will present an efficient computational scheme, combining data mining and high-throughput ab initio simulations, to identify the most stable structures and compositions among large pools of candidate systems (Section 3.4). Finally, we will discuss perspectives to apply high-throughput ab initio calculations to the research of alkali antimonides and tellurides, in order to address most efficiently the challenges outlined in Figure 1.

## 2. Methods

### 2.1. Theoretical Background

Density-functional theory (DFT) is the flagship method for ab initio electronic structure theory. Its foundation dates back to the work of Walter Kohn and collaborators published in the mid 1960s [71,72,73]. The key idea behind DFT is to express all the observables related to a many-body system as a functional of its electron density [71]:

(1) $$n\left(r\right)=\sum_{i=1}^{occ.}{\left|\psi_{i}\left(r\right)\right|}^{2},$$

where the sum runs over all occupied states. This approach is particularly convenient, as it enables to shift the focus from determining the total wave-function of the *N*-electron system—which is an impossible task—to access its electron density only. The single-particle wave-functions appearing in Equation (1) can be computed as the solutions of the so-called Kohn–Sham (KS) equations (in atomic units, adopted hereafter):

(2) $$\left[-\frac{\nabla^{2}}{2}+v_{s}\left(r\right)\right]\varphi_{ik}\left(r\right)=\epsilon_{ik}^{KS}\varphi_{ik}\left(r\right).$$

These are a set of Schrödinger-like equations mapping the many-electron problem into an auxiliary framework of non-interacting particles [72], described by the states $\varphi_{ik}$, where the subscripts *i* and k indicate the energy level and the k-vector, respectively. The eigenvalues of Equation (2) represent the single-particle energies of this fictitious system, and their physical significance has to be treated with care [74,75]. The Hamiltonian in Equation (2) contains the usual kinetic-energy term and the effective KS potential, which is the sum of three contributions: $v_{s}\left(r\right)=v_{ext}\left(r\right)+v_{H}\left[n\right]\left(r\right)+v_{xc}\left[n\right]\left(r\right)$. The external potential $v_{ext}$ accounts for the Coulomb attraction between electrons and nuclei; the Hartree potential, $v_{H}\left[n\right]\left(r\right)$, describes the electron–electron Coulomb repulsion in a mean-field fashion, and the exchange correlation (xc) potential, $v_{xc}\left[n\right]\left(r\right)$, includes the remaining particle–particle interactions. This term is the only one that needs to be approximated, as its exact form is unknown. The simplest, local density approximation (LDA) consists of treating exchange and correlation as in the homogeneous electron gas [72]. The so-called generalized gradient approximation (GGA) [76], which became popular with the successful Perdew–Burke–Ernzerhof (PBE) implementation [77], represents the next rank of sophistication. Higher-level approximations that are currently applicable to solid-state systems for up to several hundreds of atoms include metaGGA functionals (Tran-Blaha [78] and SCAN [79] are among the most popular implementations) as well as range-separated hybrid functionals, such as HSE [80]. The inclusion of these xc potentials in DFT enables unprecedented levels of accuracy, especially in predicting electronic gaps, effective masses, and optical absorption energies [81,82,83,84,85,86]. For details about these functionals and their implementation in the various DFT packages, we refer interested readers to the original works cited above.

Although these advanced functionals extend de facto the applicability of DFT also beyond ground-state properties, state-of-the-art approaches for describing charged and neutral excitations in solids belong to the field of the many-body perturbation theory (MBPT) [87]. Even though this formalism was developed independently from DFT [88], the first attempts to connect the two methods date back to the 1980s [89,90,91]. These schemes are based on the GW approximation for the electronic self-energy ∑=GW, where *G* is the single-particle Green’s function,

(3) $$G\left(r,r^{\prime },\omega \right)=\sum_{i}\frac{\varphi_{ik}\left(r\right)\varphi_{ik}^{*}\left(r^{\prime }\right)}{\omega -\epsilon_{ik}-i\eta },$$

including KS states and energies from Equation (2), and η is an infinitesimal positive number. *W* is the dynamically screened Coulomb interaction,

(4) $$W\left(r,r^{\prime },\omega \right)=\int \varepsilon^{-1}\left(r,r_{1},\omega \right)\;v_{C}\left(r_{1},r^{\prime }\right)\;dr^{\prime },$$

where $\varepsilon^{-1}$ is the frequency-dependent dielectric function and $v_{C}$ the bare Coulomb potential. Both *G* and *W* can be determined self-consistently. However, in practice, the perturbative, non-self-consistent approach $G_{0}W_{0}$ is most commonly used. In this scheme, also adopted herein, both *G* and *W* are computed “single-shot” on top of DFT. The self-energy evaluated from GW enters the quasi-particle (QP) equation, which in the $G_{0}W_{0}$ scheme reads as follows [92]:

(5) ϵikQP=ϵikKS+ZikRe∑ik(ϵikKS)−vikxc,

where $\epsilon_{ik}^{QP}$ are the QP energies, $v_{ik}^{xc}$ is the xc potential from DFT, and $Z_{ik}$ is the renormalization factor accounting for the energy-dependence of the self-energy.

Optical excitations are computed on top of the QP band structure from the solution of the Bethe–Salpeter equation (BSE) [93], which is the equation of motion for the two-particle electron-hole Green’s function [94]. The BSE formalism can be applied also to the calculation of core-level excitations. This is straightforward in the all-electron implementations of DFT and MBPT where core electrons are explicitly accounted for [95,96,97]. In this scenario, the GW step is typically omitted, and the QP correction to the gap is included in the applied scissors operator [96,98,99,100,101,102,103,104]. Either way, the BSE is cast into the effective Schrödinger-like equation as follows:

(6) $$\sum_{o^{\prime }u^{\prime }k^{\prime }}{\hat{H}}_{ouk,o^{\prime }u^{\prime }k^{\prime }}^{BSE}A_{o^{\prime }u^{\prime }k^{\prime }}^{\lambda }=E^{\lambda }A_{ouk}^{\lambda },$$

where *o* and *u* label initial occupied and final unoccupied states, respectively. In spin-unpolarized systems, the effective two-particle Hamiltonian [105], ${\hat{H}}^{BSE}$, is expressed as follows:

(7) $${\hat{H}}^{BSE}={\hat{H}}^{diag}+{\hat{H}}^{dir}+2{\hat{H}}^{x}.$$

The *diagonal* term, ${\hat{H}}^{diag}$, accounts only for the energy differences between occupied and unoccupied states; ${\hat{H}}^{dir}$ corresponds to the *direct* Coulomb attraction between the positively-charged hole and the negatively-charged electron, as follows:

(8) $${\hat{H}}^{dir}\;\;=\;-\;\;\int \;\;\;d^{3}r\;\;\int \;\;d^{3}r^{\prime }\phi_{ok}\left(r\right)\phi_{uk}^{*}\left(r^{\prime }\right)W\left(r,r^{\prime }\right)\phi_{o^{\prime }k^{\prime }}^{*}\left(r\right)\phi_{u^{\prime }k^{\prime }}\left(r^{\prime }\right).$$

where the integral is ruled by the screened Coulomb interaction $W=\varepsilon^{-1}v_{C}$, with ε being the static dielectric screening tensor of the system. The third term in Equation (7) includes the *exchange* interaction between the electron and the hole:

(9) $${\hat{H}}^{x}=\int d^{3}r\int d^{3}r^{\prime }\phi_{ok}\left(r\right)\phi_{uk}^{*}\left(r\right)\bar{v}\left(r,r^{\prime }\right)\phi_{o^{\prime }k^{\prime }}^{*}\left(r^{\prime }\right)\phi_{u^{\prime }k^{\prime }}\left(r^{\prime }\right),$$

where $\bar{v}$ is the short-range part of the bare Coulomb potential accounting for local-field effects [106,107].

The eigenvalues $E^{\lambda }$ in Equation (7) represent the excitation energies. Exciton binding energies ($E_{b}$) are typically estimated as the difference between excitation energies and the QP optical (i.e., direct) gap: $E_{b}=E^{\lambda }-E_{gap}^{QP-opt}$ [105]. This definition is appropriate for optical excitations in inorganic solids, where excitons typically manifest themselves in the form of intragap sharp resonances. However, in X-ray absorption spectra, where the onset does not coincide with the optical gap, a more accurate definition of the exciton binding energy as $E_{b}=E_{BSE}^{\lambda }-E_{IQPA}^{\lambda }$ can be adopted [67,96,104]: the second term on the right-hand side corresponds to the excitation energy calculated in the independent QP approximation (IQPA) where only ${\hat{H}}^{diag}$ is included in the effective BSE Hamiltonian (Equation (7)).

The eigenvectors of Equation (6), $A^{\lambda }$ contain information about the excited states, and weight the matrix elements in the transition coefficients,

(10) $$t^{\lambda }=\sum_{ouk}A_{ouk}^{\lambda }\frac{\langle ok|\hat{p}|uk\rangle }{\varepsilon_{uk}^{QP}-\varepsilon_{ok}^{QP}},$$

entering the expression of the imaginary part of the macroscopic dielectric function,

(11) $$ℑ\varepsilon_{M}=\frac{8\pi^{2}}{\Omega }\sum_{\lambda }{\left|t^{\lambda }\right|}^{2}\delta \left(\omega -E^{\lambda }\right),$$

which is a dimensionless quantity, where Ω is the unit cell volume and ω the energy of the impinging photon; Equation (11) is typically adopted to describe optical or X-ray absorption.

### 2.2. Computational Costs

After reviewing the theoretical background, it is useful to briefly discuss the computational costs of the methods illustrated above. While the actual performance of the calculations depends on the specific implementations and the adopted parallelization schemes, as well as on the available hardware, it is possible to provide general indications that are useful to estimate the necessary resources. Considering systems described by up to 50 atoms in the unit cell, DFT calculations with semi-local functionals based on the LDA or the GGA can be performed nowadays within a few hours on standard desktop workstations with 16 computing cores and 32 GB of memory. The choice of metaGGA functionals does not dramatically impact on these costs, increasing them up to ∼20–30%, depending on the system. On the other hand, adopting hybrid functionals, such as HSE06, demands more time and computing power, due to the calculation of the Fock exchange integral and of the dielectric screening tensor; in this case, computational costs can increase by a factor of 100 or more, compared to LDA or GGA calculations. In addition to the choice of the exchange-correlation functional, also the basis set and the treatment of core electrons are expected to play a role. Plane–wave basis sets are certainly very convenient to treat periodic crystals. However, efficient implementations of localized basis sets can also be practically employed in these calculations at comparable computational costs to plane waves [108,109,110]. Pseudopotentials offer an additional way to simplify DFT calculations at the expense of losing information about core electrons. Although generally more expensive than pseudopotential implementations, modern all-electron codes [111,112,113] provide an excellent trade-off between accuracy and computational costs.

Performing MBPT calculations entails significantly higher computing power and time. Responsible for this are the non-local operators that are present at both the GW and the BSE level. In GW runs, the numerical bottleneck is the evaluation of the dynamically screened Coulomb interaction *W* (Equation (4)), while in the BSE, it is in the calculation of the direct and exchange Coulomb integrals (Equations (8) and (9)). For these reasons, MBPT calculations typically require computing architectures with a much larger number of nodes and larger memory. Efficient numerical schemes implemented in several codes [114,115,116] have partly alleviated these problems, enabling a significant speed-up on highly parallelized hardware. All in all, at present, MBPT calculations (GW and BSE) on systems with about 50 atoms are performed on the timescale of the order of a few tens of hours.

## 3. Results and Discussion

### 3.1. Electronic Structure

**Figure 2:**
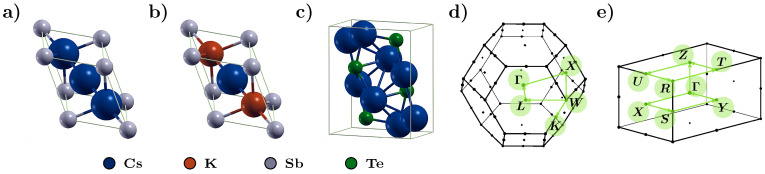
Ball-and-stick representation of the primitive unit cells of (**a**) Cs${}_{3}$Sb, (**b**) CsK${}_{2}$Sb, and (**c**) Cs${}_{2}$Te. Brillouin zone of (**d**) Cs${}_{3}$Sb and CsK${}_{2}$Sb and (**e**) Cs${}_{2}$Te with the high-symmetry points and the paths connecting them being highlighted in green. Plots produced with the visualization software XCrysDen [122].

In the analysis of the electronic structure of Cs${}_{3}$Sb, CsK${}_{2}$Sb, and Cs${}_{2}$Te, we focus on cubic (Cs${}_{3}$Sb and CsK${}_{2}$Sb) and orthorhombic (Cs${}_{2}$Te) structures containing a minimal number of atoms in their unit cells (see Figure 2). Cs${}_{3}$Sb and CsK${}_{2}$Sb can be described in a face-centered cubic lattice with four atoms in the unit cell, according to the corresponding stoichiometry. These structures were already adopted in previous theoretical works [65,66,117,118,119] as a simplification of the cubic lattice [120], which was simulated explicitly in other computational studies [59,60,62,69,121]. As we will discuss in Section 3.4 for the case of Cs${}_{3}$Sb, this is only one of the possible crystal structures that these materials can exhibit. Nonetheless, as confirmed by the experimental findings [120], this choice is fully justified. Importantly, the small size of considered unit cells enables an accurate description of their electronic and optical properties via MBPT.

**Figure 3:**
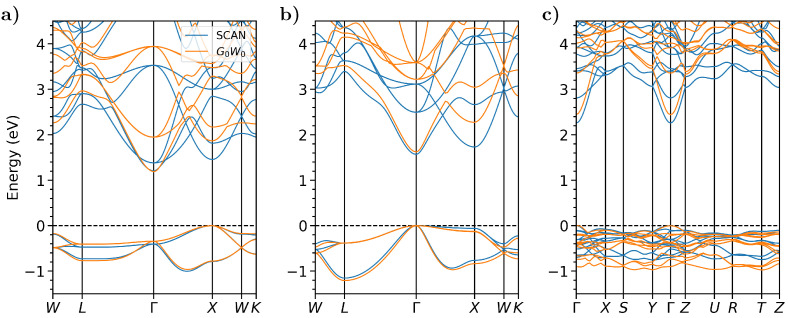
Electronic band structures of (**a**) Cs${}_{3}$Sb, (**b**) CsK${}_{2}$Sb, and (**c**) Cs${}_{2}$Te calculated from DFT with the SCAN functional (blue) as well as from *G*${}_{0}$*W*${}_{0}$ on top of PBE (orange). The zero of the plots is set to the top of the valence band of both calculations. The data for the SCAN band structures of Cs${}_{3}$Sb and Cs${}_{2}$Te coincide with those published in Reference [70].

We start our discussion from band structures computed from DFT using the SCAN functional and from $G_{0}W_{0}$ on top of PBE (see Figure 3). For convenience, in all plots, the bands calculated with the two methods are aligned at the valence band maximum (VBM) set to 0 eV. We notice that for the three materials, the band gaps featured by SCAN and $G_{0}W_{0}@$PBE are in excellent agreement with each other (see also Table 1). The success of the SCAN functional in describing the band gaps of these materials offers a computationally efficient and yet reliable alternative to the much more costly GW calculations [70].

A careful inspection of the band structures in Figure 3 reveals, however, some differences in the results provided by these two methods. In the conduction region of the three materials, the minima of the bands just above the lowest-energy one are systematically downshifted by SCAN, compared to $G_{0}W_{0}$. In particular, in Cs${}_{3}$Sb, this changes the nature of the optical gap, which is predicted to be at X by SCAN, while at Γ in $G_{0}W_{0}@$PBE (see Table 1). Comparing the band-gap values reported in Table 1, we indeed notice a systematic reduction in the difference between the electronic and the optical gap obtained from PBE, where it amounts to 370 meV, and from SCAN, where it decreases to 250 meV. The results given by the $G_{0}W_{0}@$PBE calculation preserve approximately the difference between the electronic and the optical gap as in the PBE calculation. In Cs${}_{2}$Te, a similar effect is present also on the bottom of the valence region (see Figure 3c): in this case, the SCAN result yields a reduced width of this band manifold by approximately 400 meV, compared to $G_{0}W_{0}@$PBE. Nonetheless, the band dispersion is reproduced almost identically by SCAN and by $G_{0}W_{0}@$PBE, thereby hinting to the fact the two methods will also provide similar values of the effective masses.

**Figure 4:**
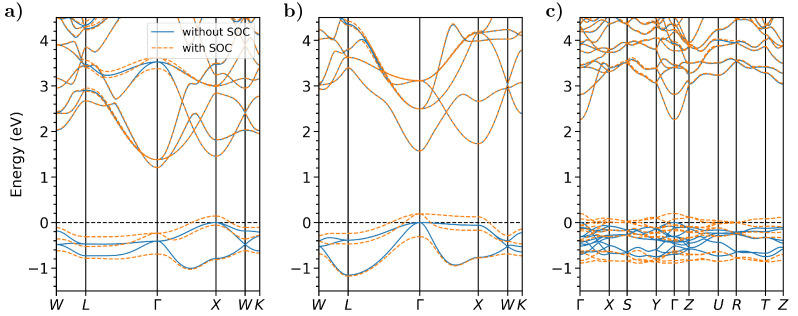
Electronic band structures of (**a**) Cs${}_{3}$Sb, (**b**) CsK${}_{2}$Sb, and (**c**) Cs${}_{2}$Te calculated from DFT (SCAN functional), with and without including spin-orbit coupling (blue solid lines and orange dashed lines, respectively). The energy range is offset with respect to the valence band maximum in the calculation without spin-orbit coupling. The data for Cs${}_{3}$Sb and Cs${}_{2}$Te coincide with those published in Reference [70].

Another important effect to take into account in the calculation of the band structure of these materials is spin-orbit coupling (SOC). It is indeed known that such relativistic effects are often non-negligible in the presence of heavy atomic species, such as Cs and Sb. As shown in Figure 4 for DFT calculations with the SCAN functional, in all three materials, SOC leads to a splitting of the valence bands, which are dominated by Sb *p*-states [65,70]. Notice that the character of these bands and the corresponding spin-orbit splittings (see below) do not change significantly when Cs is replaced by Na [123]. The SOC-induced splittings up-shift the energy of the VBM by a few hundred meV (see Figure 4). No significant changes are noticed in the conduction region. This is consistent with the fact that the lowest unoccupied bands are dominated by *s*-states [65,66,70], which are not subject to this effect. The only exception is in the band structure of Cs${}_{3}$Sb, where a splitting appears in the vicinity of Γ at approximately 3.5 eV above the VBM. As reported in Refs. [65,66,70], this band is dominated by Cs *d*-states. Upon inclusion of SOC, the band gaps decrease by about 150 meV in both Cs${}_{3}$Sb and CsK${}_{2}$Sb and by 200 meV in Cs${}_{2}$Te (see Table 1). These results suggest that in these materials, SOC is indeed non-negligible. However, being aware of the magnitude of the spin-orbit splitting and knowing that it is almost equal in the compounds of this family enables one to estimate the impact of this effect, even when its implicit inclusion in the calculations is numerically unfeasible. Indeed, relativistic corrections further increase the computational costs of MBPT calculations (see Section 2.2), making them almost prohibitive for real materials. Recent attempts to include SOC in MBPT runs were successfully realized making use of a simplified version of the BSE [124].

### 3.2. Optical Spectroscopy

**Figure 5:**
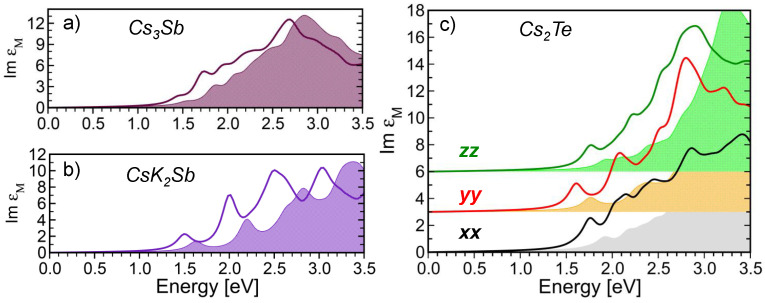
Optical absorption spectra of (**a**) Cs${}_{3}$Sb, (**b**) CsK${}_{2}$Sb, and (**c**) Cs${}_{2}$Te calculated, including and neglecting excitonic effects, by solving the full BSE (solid lines) or in the IQPA (shaded areas), respectively. In the spectra of Cs${}_{3}$Sb and CsK${}_{2}$Sb, all diagonal components of the dielectric tensor are equivalent. A Lorentzian broadening of 100 meV is applied to all spectra to mimic the excitation lifetime. The data reported in panel (**b**) coincide with those published in Reference [65].

With the insight gained from the analysis of the band structures, we inspect now the optical spectra of Cs${}_{3}$Sb, CsK${}_{2}$Sb, and Cs${}_{2}$Te computed from the solution of the BSE on top of $G_{0}W_{0}@$PBE (see Figure 5). In all three systems, the absorption onset appears at the boundary of the visible region, around 1.5 eV (see also Table 1). Comparing the BSE results with the experimental references reported in Table 1, we notice for Cs${}_{3}$Sb a very good agreement between our calculated result (1.44 eV) and the value of 1.6 eV measured by Spicer in the 1950s [38]. In the other two materials, differences with respect to experimental references are more pronounced. Specifically, in CsK${}_{2}$Sb, the BSE optical gap overestimates the measurement by Ghosh and Varma [10] by 300 meV. In Cs${}_{2}$Te, the experimental value [39] is twice as large as the theoretical value.

We can elaborate on this remarkable discrepancy by examining in more detail the optical spectrum shown in Figure 5c. In comparison with the antimonide materials, which are considered here in their cubic crystal structure, orthorhombic Cs${}_{2}$Te is characterized by three inequivalent, diagonal components of the dielectric tensor. The absorption onset is along yy, followed by the maxima in the xx and zz components. In the spectral window shown in Figure 5c, the oscillator strength reaches its maximum between 2.5 eV and 3.0 eV, with a relative intensity that is approximately three times larger, compared to the one at the onset. This behavior can be understood considering the relatively low joint density of states associated to the transitions from the highest-occupied *p*-bands and the lowest-unoccupied *s*-bands. However, while in both Cs${}_{3}$Sb and CsK${}_{2}$Sb, the lowest-energy excitation is bright [65], in Cs${}_{2}$Te, the first four excitations are so weak that they do not contribute to the absorption. The lowest-energy peak in the yy component of the dielectric tensor corresponds to the fifth excited state. Considering that the reference measurements in Ref. [39] correspond to the quantum yield, it is legitimate to speculate that higher-energy maxima in the absorption spectrum contribute to the first step of the photoemission process that is finally probed in experiments. While this analysis does not provide an ultimate clarification of the discrepancy between the experimental data and MPBT calculations, it certainly shows the complexity of this material and hopefully stimulates dedicated work to decipher its photo-physics. The coexistence of crystal polymorphs, the presence of defects, and electron–phonon coupling are among the most relevant effects that should be investigated to solve this conundrum.

We conclude this discussion by analyzing the excitonic effects in the spectra shown in Figure 5. In addition to the results from BSE (solid lines), which were examined so far, the spectra computed in the IQPA are also displayed (shaded areas). The latter provide analogous information to the joint density of states. Comparing for each material the results of these two calculations in which excitonic effects are included (BSE) and excluded (IQPA), it is evident that electron-hole couplings do not generate any new absorption resonance. This is in contrast to the known features of conventional semiconductors [125,126] and insulators [97,127,128]. The reason behind this behavior of Cs${}_{3}$Sb, CsK${}_{2}$Sb, and Cs${}_{2}$Te is related to the relatively low values of their static dielectric permittivities. Evaluated within the random phase approximation, they are $\varepsilon_{r}$ = 8.96 for Cs${}_{3}$Sb, $\varepsilon_{r}$ = 6.40 for CsK${}_{2}$Sb, and $\varepsilon_{r}$ = 5.76 for Cs${}_{2}$Te. These values indicate that screening in these materials is not very effective and even in the BSE spectra, the absorption peaks at the onset retain their character of band-to-band transitions [65]. A similar behavior was found also in the optical spectra of Na-based potassium antimonides [123]. Hence, in these materials, excitonic effects manifest themselves only as a redistribution of spectral weight to lower energies. This energy shift is a consequence of the inclusion of the attractive electron-hole interaction in the BSE calculation (Equation (8)), which dominates over the exchange term (Equation (9)). This is not the case, for example, in organic materials, where local field effects ruled by the electron-hole exchange are crucial [129]. Comparing the energy of the absorption maxima obtained in the BSE and in the IQPA calculations, we can estimate binding energies for the lowest-energy excitations in the order of a few hundreds of meV.

### 3.3. Core-Level Spectroscopy

As anticipated in Section 2, the BSE formalism can be efficiently exploited also to calculate X-ray absorption spectra in the adopted all-electron framework of MBPT [96,97]. In a dedicated study, we recently investigated the X-ray absorption fingerprints of inequivalent Cs atoms in Cs${}_{3}$Sb [67] considered in the same geometry depicted in Figure 2a. In that work, we noticed that the local environment of the individual Cs atoms strongly impacts the X-ray spectral features. This is expected based on the knowledge over even more complex systems, such as Ga${}_{2}$O${}_{3}$, where crystallographically inequivalent atoms are also chemically inequivalent and exhibit different coordination [103,130]. In Ref. [67], we also found remarkable excitonic effects in the X-ray absorption spectra of Cs${}_{3}$Sb from the $L_{3}$-edge, which are featured also by CsK${}_{2}$Sb [65].

**Figure 6:**
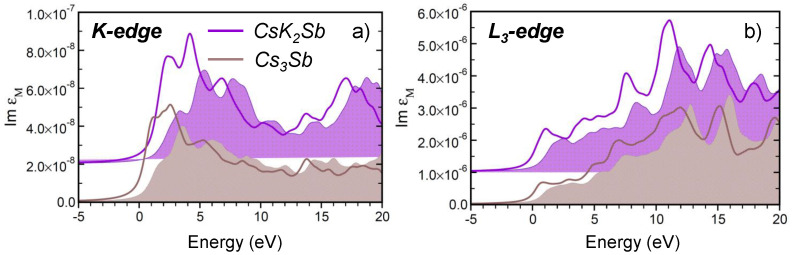
X-ray absorption spectra from (**a**) the Sb *K*-edge and (**b**) the Sb $L_{3}$-edge of Cs${}_{3}$Sb and CsK${}_{2}$Sb, calculated by including and neglecting excitonic effects by solving the full BSE (solid lines) or in the IQPA (shaded areas), respectively. The edge of the IQPA spectra is set to 0 eV, and the BSE results are aligned to it accordingly. A Lorentzian broadening of 500 meV is applied to all spectra to mimic the excitation lifetime.

Here, we discuss the computed X-ray absorption spectra of Cs${}_{3}$Sb and CsK${}_{2}$Sb obtained by exciting the 1s ad $2p_{3/2}$ electrons of Sb, which correspond to the *K*-edge and the $L_{3}$-edge, respectively. Notice that we can decouple the $L_{3}$- from the $L_{2}$-edge, due to the huge spin-orbit splitting (>200 eV) between the Sb $2p_{3/2}$ and $2p_{1/2}$ core electrons in both materials [66]. Since there is only one Sb atom in both considered crystals (see Figure 2a,b) there are no inequivalent contributions to take into account in this case. The results displayed in Figure 6 are plotted with the zero of the energy scale coinciding with the onset of the spectra computed in the IQPA, where electron-hole couplings are neglected.

We start our analysis with the spectra computed from the Sb *K*-edge of Cs${}_{3}$Sb and CsK${}_{2}$Sb (see Figure 6a). In both materials, a broad peak appears at the onset with the second maximum being more intense than the first one. The comparison between the BSE and IQPA results indicates that, similar to the optical spectra described in the previous section, excitonic effects act mainly on these first peaks by red-shifting their energies on the order of a few hundreds of meV and by enhancing their oscillator strength. Notice that, in contrast, excitonic effects are particularly pronounced in the X-ray absorption spectra from the Cs *K*-edge in Cs${}_{3}$Sb [67]. Comparing now the spectra computed for the two materials (Figure 6a), we do not notice any peculiar signatures that would enable their identification in samples where they coexist. This similarity can be explained by the almost identical chemical environment of the Sb atom in the two materials.

The X-ray absorption spectra from the Sb $L_{3}$-edge are also remarkably similar in Cs${}_{3}$Sb and CsK${}_{2}$Sb (see Figure 6b) and, as such, they do not provide any fingerprints to pinpoint the presence of either material in mixed samples. X-ray photoemission spectroscopy is certainly more effective for this purpose [66]. In the spectra from the Sb $L_{3}$-edge, the spectral weight grows from the onset to higher-energies. This behavior can be understood considering that, according to the Δl=±1 selection rule, target states of transitions from the Sb $2p_{3/2}$ states are conduction bands with both *s* and *d* characters. At the onset of the spectra shown in Figure 6b, transitions to the *s*-like lowest unoccupied bands dominate: due to the characteristic parabolic dispersion of these states, the associated density of the states is relatively weak [65,67]. Starting at approximately above 5 eV from the onset up to higher energies, transitions to unoccupied *d* states start contributing more significantly to the absorption. These bands give rise to a larger density of states, which, in turn, explains the larger oscillator strength in the X-ray absorption spectra. Notice that, because of the dominant *s* and *d* characters of the lowest conduction bands, the relative peak intensities in the $L_{3}$-edge spectra are approximately two orders of magnitude larger, compared to the spectra from the *K*-edge. Excitonic effects in the $L_{3}$-edge spectra are even less prominent than in the *K*-edge spectra. In this case, electron-hole couplings induce merely an almost rigid red-shift of the spectral features by a few hundreds of meV.

### 3.4. High-Throughput Material Screening

In parallel with the development and the application of advanced theoretical methods, such as MBPT, which enable an accurate description of the excited-state properties of complex materials, another line of research that recently emerged in computational materials science is high-throughout screening based on DFT [47]. This term describes extensive numerical studies, exploring a large number of compounds in an initially selected pool and filtering them according to the desired properties. A distinct characteristic of these calculations is automatization: a computational workflow rules all the involved steps, including input preparation, execution, and output parsing. Based on these general principles and their various implementations in different libraries and software packages [131,132,133], over the last decade, a number of open-access computational databases have been established, such as AFLOW [134], the Open Quantum Materials Database [135], the Materials Project [136], NOMAD [137], and Materials Cloud [138]. These facilities give access to the properties of hundreds of thousands of different crystal structures calculated from DFT, thereby opening unprecedented opportunities in the field of materials science. Data-mining approaches can be used to identify new promising candidate materials or material classes for specific applications [139,140,141], and machine learning algorithms can be applied to the existing data to predict properties of unknown materials [142,143]. Here, we illustrate our first steps in this direction, utilizing the resources of shared online databases. As an example, we identify stable stoichiometric phases of binary cesium-antimonide materials and determine their electronic gap.

**Figure 7:**
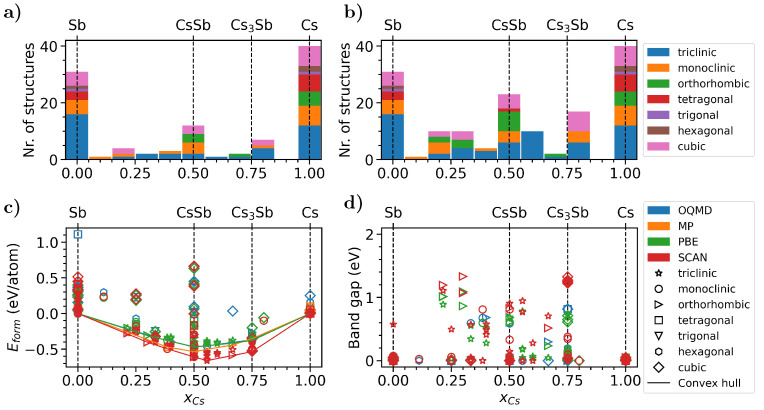
Histogram of the number of cesium–antimonide structures (**a**) mined directly from MP and OQMD and (**b**) generated from chemically analogous structures upon exchange of anions and cations (details in the text); (**c**) formation energy ($E_{form}$), including the convex hull, and (**d**) band gap of the identified structures depending on the concentration of Cs, as taken directly from the databases (OQMD and MP) and recalculated in this work with the PBE and SCAN functionals (see color and symbol codes in the legend).

We start off by mining the online databases Materials Project (MP) [136] and Open Quantum Materials Database (OQMD) [135] to identify all available crystal structures containing exclusively Cs and Sb elements. For this task, we employ an in-house developed Python script. From this search, 34 and 94 systems emerge from MP and OQMD, respectively. All these structures are compared against each other, using the F-fingerprint method developed by Oganov and Valle [144] in order to eliminate duplicates. This procedure leads to a pool of 117 crystal structures in total. Since most databases rely on experimental crystal structures, i.e., materials registered in the ICSD database [145,146], phases are often unevenly distributed throughout the relevant compositional range. In the examined case, there is a very large fraction (∼68%) of structures consisting of elemental phases; see Figure 7a. To reduce the weight of these compounds in our initial pool, we extend the database search to binary structures containing K, Rb or Cs as cations and As, Sb or Bi as anions, which are chemically similar to our target compounds. After the identification of these additional materials in both MP and OQMD, we exchange the cation and anion with Cs and Sb, respectively, concomitantly rescaling the lattice parameters and the atomic positions, according to the mean of the ratio of the covalent radii of the substituted elements. In this way, the pool of candidate materials is increased to 170 crystal structures, thereby reducing the relative amount of elemental phases to 47%; see Figure 7b.

After establishing the initial set of crystal structures, we proceed with the high-throughput calculations. The first step consists of the determination of the space group of each crystal using the library spglib [147]. Next, lattice vectors and atomic positions of each material are optimized until the interatomic forces are smaller than 0.01 eV/Å. During this step, a constraint is applied to preserve the pre-assigned space group for each crystal. At the end of this optimization, all structures are compared against each other once again, using the previously described method for discarding duplicates. Next, DFT calculations are performed on each system to determine their formation energy and electronic band gap. The former is computed as the difference between the energies per atom of the binary compounds [$E({\mathrm{Cs}}_{x}{\mathrm{Sb}}_{1-x})$] and those of the most stable elemental phases of the constituents [E(Cs) and E(Sb)]:

(12) $$E_{form}\left({\mathrm{Cs}}_{x}{\mathrm{Sb}}_{1-x}\right)=E\left({\mathrm{Cs}}_{x}{\mathrm{Sb}}_{1-x}\right)-\left[xE\left(\mathrm{Cs}\right)+\left(1-x\right)E\left(\mathrm{Sb}\right)\right].$$

Negative values of $E_{form}$ are indicative of stable compounds. Total energies computed from DFT are used in Equation (12). Hence, neither thermal effects nor zero-point vibrational contributions are accounted for. The same workflow is applied using both the PBE and SCAN functionals (for details of these calculations, see Appendix A). While PBE is routinely used for high-throughput calculations, due to its popularity and its low computational costs, here, we additionally employ SCAN due to its proven superior performances in the description of the electronic structure of cesium antimonides, comparable with MBPT on top of PBE (see Figure 3 and Ref. [70]).

The phase diagrams with the formation energies computed for the selected materials are presented in Figure 7c together with the results stored in the OQMD and MP databases [135,136], the latter being computed with the software package VASP [148] employing the PBE functional. It can be immediately seen that only part of the examined compounds has negative formation energies and, therefore, can be considered stable. Comparing first the convex hull obtained from the entries of the two databases, a remarkable difference of 0.07 eV/atom is noticed at the chemical composition CsSb ($x_{Cs}$ = 0.50) with the results from OQMD being lower in energy. Toward Cs-rich compositions (left side of Figure 7c) this trend is reverted, with the data from MP being slightly higher in energy for Cs${}_{3}$Sb ($x_{Cs}$ = 0.75). The reason for this behavior can be found in the empirical correction scheme applied on the data originating from MP [149]. Additionally, the Cs phase corresponding to the lowest energy is cubic, while in the OQMD, it is trigonal and, evidently, more stable.

Our results obtained with the PBE functional match well with the ones in the OQMD and show qualitative agreement with data from MP, given the discrepancy discussed above. The minimum of the convex hull resulting from our PBE results is situated at $x_{Cs}$ = 0.50, where the formation energy amounts to 0.47 eV/atom; the lowest formation energy for the Cs${}_{3}$Sb composition ($x_{Cs}$ = 0.75) is 0.38 eV. Additional stable structures are obtained for the chemical formulas Cs_3_Sb_7_, Cs_4_Sb_2_, and Cs_5_Sb_8_, in agreement with data reported in the ICSD. While the last two phases are present in both MP and OQMD, the first one was added to our pool of structures through the search of chemically similar compounds, as explained above. Comparing now these outcomes with the convex hull obtained with SCAN, we notice a clear tendency toward lower formation energies obtained with the latter functional. This finding is not unexpected, considering the superior behavior of this metaGGA implementation in predicting cohesive and formation energies in better agreement with experiments compared to PBE [150,151]. For this set of data, we find the minimum of the convex hull shifted to a ratio of 5:4 ($x_{Cs}$ = 0.56) with a formation energy of 0.66 eV/atom. Interestingly, this structure corresponds to a novel phase with space group P1 that is generated again via chemical similarity. These qualitative differences between the results obtained with PBE and SCAN highlight the importance of choosing advanced exchange-correlation functionals in the treatment of these materials, even for the description of ground-state properties, such as formation energies. In terms of computed band gaps, a variety of metallic and semiconducting phases appears from the plot in Figure 7d. As expected, the results obtained for this quantity with the SCAN functional are generally larger than those from PBE, due to its more accurate treatment of the exchange-correlation potential.

Given its relevance in experimental works [22,152], it is worth deepening the analysis on Cs${}_{3}$Sb. The face-centered cubic crystal structure (space group $Fm\bar{3}m$) associated with this composition and also considered above in the analysis of spectroscopic properties calculated from MBPT features a formation energy of −0.35 eV/atom computed from PBE, situating this phase 0.027 eV/atom above the convex hull shown in Figure 7c. Using the SCAN functional, the formation energy reduces to −0.53 eV/atom, subsequently also reducing the difference to 0.006 eV/atom. Cubic structures with space group $Fd\bar{3}m$ are not found for Cs_3_Sb in the mined databases. On the other hand, in the same pool of data, a number of monoclinic and triclinic structures for the Cs${}_{3}$Sb stroichiometry with formation energies below a threshold of 0.02 eV/atom above the convex hull are found for both PBE and SCAN functionals. For Cs${}_{3}$Sb, we find values for the band gap ranging from 0.0 eV up to 0.73 eV from PBE, and up to 1.33 eV from SCAN. Among the most stable phases, the triclinic and monoclinic structures exhibit band gaps between 0.09–0.16 eV and 0.35–1.26 eV, respectively; the face-centered cubic phase is characterized by a band gap of 0.73 eV according to PBE, and of 1.29 eV from SCAN.

The high-throughput analysis presented above shows the potential of this novel computational approach to explore the vast configurational space of photocathode materials. The promising results predicted by earlier theoretical works [58,62,117,119,121,123] suggest favorable properties also for alkali antimonides with lighter species than Cs. Including both binary and ternary phases dramatically enhances the amount of candidate systems, making high-throughput calculations the most efficient and feasible way to explore their properties. Additionally, the research on telluride compounds is expected to greatly benefit from this approach. As discussed in Section 3.2, fundamental questions regarding the photo-physics of Cs${}_{2}$Te are still unanswered and exploring the configurational space of its stable crystal structures as well as of other stoichiometries is essential to extend the current knowledge. The approach illustrated herein with the examples of formation energies and band gaps can be extended to the inclusion of thermal properties that are certainly crucial for photocathodes in operational conditions. This type of analysis is also prone to promising interfaces with machine learning approaches.

## 4. Conclusions and Outlook

We have presented an overview of the capabilities of the state-of-the-art first-principles methods to investigate the microscopic properties of cesium-based photocathode materials. Even with semi-local functionals, such as PBE, DFT provides a qualitatively correct picture of the electronic structure of the considered antimonides and telluride materials, as demonstrated herein for the cubic crystals Cs${}_{3}$Sb and Cs${}_{2}$KSb, as well as for orthorhombic Cs${}_{2}$Te. Higher accuracy can be achieved either in the framework of the many-body perturbation theory, applying the single-shot $G_{0}W_{0}$ approximation on top of the PBE results, or through a more advanced approximation of the exchange-correlation potential as provided by the SCAN functional. The second option is much more favorable in terms of computational costs and yet proven to be as reliable as the application of range-separated hybrid functionals for these material classes [70].

The optical properties of these compounds can be reliably calculated from the many-body perturbation theory through the solution of the BSE on top of the GW-corrected band structure. The all-electron implementation of DFT and MBPT provided by the exciting code employed in this work enables the calculation of optical and core excitations on the same footing [96,97]. These capabilities were demonstrated with the computed optical spectra for all the aforementioned materials as well as for the X-ray absorption spectra of the two antimonides from the Sb K- and L${}_{3}$-edges.

Finally, we have shown the application of high-throughput calculations to identify stable stoichiometries and crystal structures of Cs–Sb binary compounds. In this step, we have profited from the available DFT calculations stored in open-access databases. For the final pool of structures, filtered from the initial set by applying stability and similarity conditions, both formation energies and band gaps were analyzed in order to identify useful indications for comparison with experimental data. For example, we found that the face-centered-cubic phase of Cs${}_{3}$Sb is not predicted to be the most stable structure. While the presented analysis does not yet include thermal effects, which are crucial for the growth of these systems, it clearly suggests that, based on their energetics, several crystal structures can coexist in one sample.

The combination of the computational methods adopted in this work is extremely promising in view of complementing experiments in the search for novel and efficient semiconducting materials for photocathode applications. As mainly bulk crystals were investigated so far, detailed studies of surfaces, point defects, and grain boundaries are urgently needed to gain microscopic understanding of the microscopic mechanisms that can degrade the performance of photocathodes, in order to prevent or at least minimize them. Additionally, for these tasks, the application of the high-level many-body theory on top of a restricted pool of systems selected via automatized procedures is expected to be highly beneficial. Challenges for ab initio work in this field are not only related to the identification and characterization of optimal compositions, but they include also the prediction of the photoemission yield. The promising results obtained recently by feeding Spicer’s three-step model [37] with DFT results [46] anticipate bright perspectives in this direction. More generally, first-principles methods combined with automatized workflows elevate the role of theory to the same rank as laboratory work in the forthcoming photocathode research. The herein illustrated predictive power of these methods can be used to complement and even guide experiments in order to discover new materials with the targeted characteristic. Future interfaces with machine learning and data science routines will further solidify these interactions, finally projecting this line of research to the incoming era of artificial intelligence.

## Figures and Tables

**Table 1:** Electronic and optical band gaps of the materials calculated with different computational methods, including a comparison to experimental references. All values in eV.

	PBE	SCAN	SCAN + SOC	$G_{0}W_{0}@$PBE	BSE	Exp.
Cs${}_{3}$Sb						
$E_{gap}$	0.65	1.21	1.06	1.19		
$E_{gap}^{opt}$ (**k**-point)	1.02 (Γ)	1.46 (X)	1.31 (X)	1.53 (Γ)	1.44 (Γ)	1.6 [38]
CsK${}_{2}$Sb						
$E_{gap}$	0.88	1.57	1.38	1.62		
$E_{gap}^{opt}$ (**k**-point)	0.88 (Γ)	1.57 (Γ)	1.38 (Γ)	1.62 (Γ)	1.50 (Γ)	1.2 [10]
Cs${}_{2}$Te						
$E_{gap}$	1.76	2.26	2.06	2.45		
$E_{gap}^{opt}$ (**k**-point)	1.76 (Γ)	2.26 (Γ)	2.06 (Γ)	2.45 (Γ)	1.60 (Γ)	3.3 [39]

## Data Availability

The original data produced for this work are available in Zenodo at doi:10.5281/zenodo.5162929, record number 5162929.

## Appendix A. Computational Details

Electronic structure calculations on Cs${}_{3}$Sb, CsK${}_{2}$Sb, and Cs${}_{2}$Te with the SCAN functional are performed with the all-electron full-potential code FHI-aims [111]. The default *intermediate* settings are used for the accuracy of the basis set and the integration grids. To account for the relativistic effects of the core electrons, the zeroth-order approximation (ZORA) is applied. A **k**-mesh of 18 × 18 × 18 is employed for the cubic phases of Cs${}_{3}$Sb and CsK${}_{2}$Sb, while the first Brillouin zone of Cs${}_{2}$Te is sampled by a 12 × 6 × 6 mesh. The unit cells of each material are relaxed until the forces acting on each atom are below 0.001 eV/Å. Spin orbit coupling is accounted for in a post-scf perturbative approach, as detailed in Ref. [153].

Results obtained in the framework of MBPT are obtained with the all-electron code exciting [96,97,112], implementing the linearized augmented plane-wave plus local orbital method. The PBE functional [77] is used for all such DFT calculations serving as starting point for MBPT. In the GW calculations, the screened Coulomb potential *W* is in the random-phase approximation. For Cs${}_{2}$Te, muffin-tin (MT) spheres of radius 1.8 bohr are used for both atoms in all calculations. In the $G_{0}W_{0}$ step, a plane-wave cutoff rgkmax = 6.0 is adopted together with a **k**-mesh with 6 × 3 × 3 points and 200 empty states. In the BSE calculation for this material, carried out applying a scissors operator to reproduce the QP correction from GW, rgkmax = 8.0, a Γ-shifted **k**-mesh with 6 × 3 × 3 points, a cutoff for the local-field effects gqmax = 1.5 Ha, 100 empty states for the screening, and a transition space including 12 occupied and 25 unoccupied bands are considered.

In the description of Cs${}_{3}$Sb, MT spheres of radius 2.5 bohr are employed for both atoms together with a plane-wave cutoff rgkmax = 8.0. In the GW step, a **k**-mesh with 6 × 6 × 6 points and 200 empty states for *W* are taken. In the subsequent BSE calculation, a Γ-shifted **k**-mesh with 10 × 10 × 10 points, a cutoff for the local-field effects gqmax = 1.5 Ha, 100 empty states for the screening, and a transition space including 4 occupied and 6 unoccupied bands are adopted. In the calculations of the X-ray absorption spectra, a 6 × 6 × 6 **k**-mesh and 80 unoccupied bands, as final transition states are considered for both the *K*- and the $L_{3}$-edge. However, in the former calculation, 200 empty states and gqmax = 2.5 Ha were needed to evaluate the screening and the cutoff for the local-field effects, respectively. In the XAS calculation from the $L_{3}$-edge, 100 empty states for *W* and gqmax = 1.5 Ha are considered sufficient.

For CsK${}_{2}$Sb, MT spheres of radius 1.65 bohr are employed for all atoms together with a plane-wave cutoff rgkmax = 8.0. In the GW step, a **k**-mesh with 4 × 4 × 4 points and 200 empty states for *W* are adopted. In the subsequent BSE calculation, a Γ-shifted **k**-mesh with 8 × 8 × 8 points, a cutoff for the local-field effects gqmax = 1.5 Ha, 100 empty states for the screening, and a transition space, including 4 occupied and 15 unoccupied bands, are included. In the calculations of the X-ray absorption spectra, a 6 × 6 × 6 **k**-mesh is used for both the *K*- and the $L_{3}$-edge. In the former calculation, 200 empty states for the screening, gqmax = 2.5 Ha for the cutoff of local-field effects, and 100 empty target bands are included. In the XAS calculation from the $L_{3}$-edge, 100 empty states for *W*, gqmax = 1.5 Ha, and 80 target empty states are considered.

The code cp2k [110] is used for the high-throughput calculations, utilizing the Gaussians and Plane Wave method and GTH-type pseudopotentials [154,155]. Basis set and plane-wave cutoff of 500 Ry with a relative cutoff of 70 Ry are chosen to ensure convergence. The reciprocal space is sampled with a Monkhorst–Pack mesh, resulting in a maximum distance of 0.2 Å${}^{-1}$ between adjacent points. To handle the calculations, a custom automated workflow is programmed on top of the Python library AiiDA [156,157] and its cp2k-plugin [158].
